# The feasibility and safety of deeply inserted enema tubes for acute malignant left-hemicolon obstruction: an alternative solution in developing countries

**DOI:** 10.3389/fonc.2025.1522138

**Published:** 2025-04-09

**Authors:** Xinxiang Huang, Lijuan Zheng, Huifeng Wu, Xiaomei Li, Conghua Song

**Affiliations:** ^1^ Gastrointestinal Endoscopy Center, The Affiliated Hospital of Putian University, Putian, Fujian, China; ^2^ School of Basic Medicine, Putian University, Putian, Fujian, China; ^3^ Key Laboratory of Translational Tumor Medicine in Fujian Province, Putian University, Putian, Fujian, China

**Keywords:** tube, endoscopy, malignant obstruction, colorectal cancer, left hemicolon

## Abstract

**Background and aims:**

Disposable enema kits are commonly used for bowel preparation, with the anal tube typically positioned near the rectal ampulla. This study assesses the feasibility and safety of deeply inserting an enema tube in cases of acute malignant left-hemicolon obstruction.

**Methods:**

A retrospective analysis was conducted on 42 patients who underwent emergency endoscopic decompression via a deeply inserted enema tube for acute malignant left-hemicolon obstruction from January 2021 to September 2024 at a single center, the Endoscopy Centre of the Affiliated Hospital of Putian University. This analysis covered intubation duration, the success rate of intubation, the obstruction relief rate, as well as associated adverse events.

**Results:**

Thirty-six patients achieved successful tube placement, attaining a one-time success rate of 85.7% (36/42). Following successful intubation, the abdominal circumference decreased to a mean of (85.2 ± 3.0)% of the original value on the subsequent day (*P* < 0.01). Abdominal plain films depicted a significant reduction in both the quantity of gas-fluid levels and the maximal transverse diameter of the proximally obstructed colon [(4.5 ± 1.2) cm versus (7.4 ± 0.8) cm, P < 0.01]. Within 48 hours, C-reactive protein (CRP) levels plummeted by over 50%, and bowel sounds normalized within 2 - 5 days. During surgery, the bowel exhibited only slight or negligible dilation and edema, with no conspicuous fecal residues detected in the colonic cavity. Furthermore, no severe tube-related adverse events occurred either during or after intubation.

**Conclusion:**

The application of deeply inserted enema tubes proves to be both feasible and safe in treating acute malignant left-hemicolon obstruction, thus presenting itself as a viable alternative approach in developing countries.

## 1 Introduction

Colorectal cancer is one of the most prevalent cancers worldwide. Recent statistical reports indicate that, in China, the incidence and mortality rates of colorectal cancer rank third and fifth among all malignant tumors, respectively, with 376,000 new cases and 191,000 deaths (1). Cancer-related intestinal obstruction and its associated complications constitute significant independent risk factors that adversely affect the prognosis of patients with colorectal cancer. The incidence of colorectal cancer complicated by intestinal obstruction varies from 8% to 30%, with approximately 75% of obstruction sites located in the left-hemicolon (2).

Acute malignant left-hemicolon obstruction presents a significant challenge in clinical practice (3). Traditionally, the standard approach has been to perform an emergency left colonic resection with end colostomy (*i.e.*, the Hartmann procedure). Patients must be adequately informed and prepared for either a permanent colostomy or selective stoma closure. The mortality rate associated with this procedure can reach as high as 15-34% (4). Moreover, even among survivors, nearly 40% may require permanent colostomy maintenance (5). In recent years, intestinal stents and obstruction tubes have been used on a restricted scale in China, functioning as transitional therapies to bridge the gap between emergency and elective surgeries (6). Nevertheless, multiple constraints, including limited availability, insufficient health insurance reimbursement, exorbitant expenses, inadequate technical training, and resultant complications, have curbed the adoption and acceptance of these devices in developing countries, with China being no exception.

Disposable enema kits are frequently used for bowel preparation prior to colorectal examinations or treatments. They are notable for their accessibility, ease of operation, and low cost. Typically, the tip of the enema tube is manually placed near the rectal ampulla. Building on this conventional approach, endoscopic decompression via a deeply inserted enema tube for acute malignant left-hemicolon obstruction has been explored. This method aims to achieve a decompression effect similar to that of intestinal stents or obstruction tubes. It has been successfully put into practice, delivering satisfactory results, as detailed below.

## 2 Methods

### 2.1 Data source

Data were retrospectively retrieved from all 42 patients who underwent endoscopic decompression via a deeply inserted enema tube for acute malignant left-hemicolon obstruction at the Endoscopy Centre of the Affiliated Hospital (Group) of Putian University, spanning from January 2021 to September 2024. Medical disposable enema kits were used in accordance with their indications, and informed consent was obtained from all patients.

### 2.2 Main equipment and consumables

The following equipment and consumables were used: a gastroscope (GIF-Q260J, OLYMPUS), a colonoscope (PCF-Q260JI, OLYMPUS), an ultra-slim endoscope/transnasal endoscopy (GIF-XP170N, OLYMPUS), a zebra guidewire (ø 0.035 inch × L. 4500 mm, BOSTON), a mobile C-arm X-ray machine (PLX7100A, PUAI), and iohexol injection (20 ml: 13.56 g, Jiangsu Hengrui), among others. The medical disposable enema kit (Suzhou Macklin) comprised an enema tube (featuring two side holes at the tip, with an outer diameter ranging from 5.33 mm/16 Fr to 10.67 mm/32 Fr and a length of 250 to 500 mm), an extension tube (outer diameter: 6.3 ± 0.2 mm, length: 1000 mm), an enema bag (specification model: 1000 ± 100 ml), and a control valve.

### 2.3 Preoperative preparation

A preoperative emergency computed tomography (CT) scan of the abdomen was routinely carried out to pinpoint the site and nature of the obstruction. The subsequent procedures were initiated only after the diagnosis of malignant left-hemicolon obstruction was confirmed and the possibility of gastrointestinal perforation was ruled out. A comprehensive routine abdominal examination, together with an anal digital examination, was performed. If the fingertip touches feces or the examining finger gets stained with feces, it suggests the potential for incomplete intestinal obstruction. Based on the clinical situation, mild normal saline (37°C) could be administered anally to clean the distal colon one to three times before endoscopy.

### 2.4 Procedure

**Figure 1:**
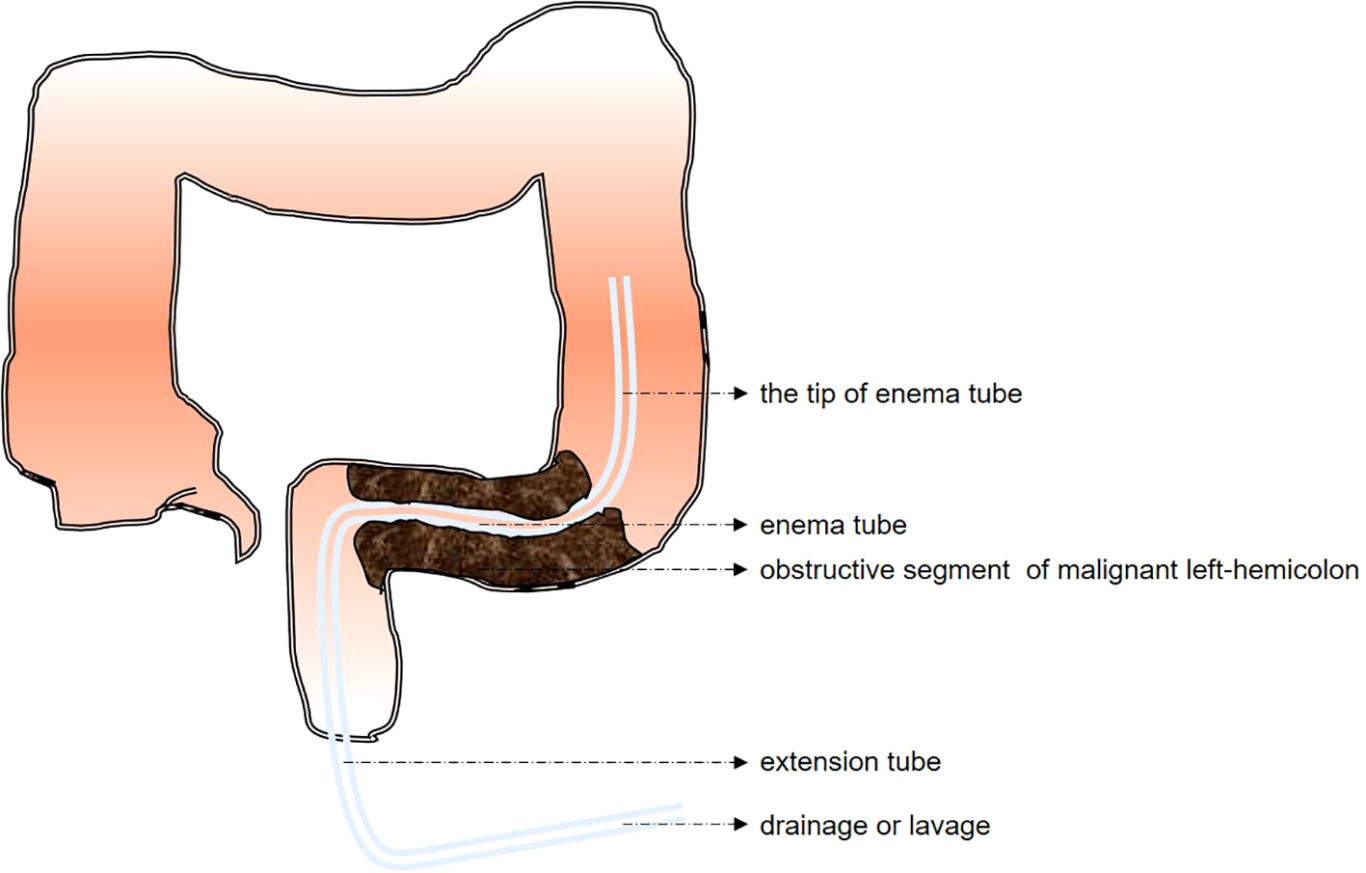
Schematic diagram of emergency endoscopic decompression using a deeply inserted enema tube for acute malignant left-hemicolon obstruction.

The procedure for endoscopic decompression via a deeply inserted enema tube for acute malignant left-hemicolon obstruction is described in detail as follows:

1. Lesion Observation: A routine endoscopic examination is carried out to locate the lesion and identify the narrow segment. Subsequently, an attempt is made to advance into the obstructive segment.
2. Determining Narrow Length: The length of the obstructive segment is measured with the endoscope. When conditions allow, the obstructive segment can be gently dilated using the endoscope. Once the endoscope has traversed the stricture, the immediate subsequent steps are suction for decompression and irrigation for drainage. These procedures are crucial for relieving the pressure within the obstructed bowel and flushing out the accumulated contents, considering the limitations and possibilities provided by the endoscopic access.
3. A guidewire is inserted approximately 50 cm beyond the oral side of the obstructive segment via the endoscopic biopsy channel, after which the endoscope is withdrawn.
4. Exceptional Situations: X-ray fluoroscopy is used to confirm the position of the distal end of the guidewire within the colonic lumen and to assess the length of the lesion when the ultra-slim endoscope cannot traverse the obstructive segment.
5. Tube Placement: The endoscope is re-inserted, and a tube of appropriate size is advanced along the guidewire under endoscopic visualization. Since the tube does not have a fixation device, its distal end within the bowel lumen is usually positioned approximately 10 - 15 cm deeper on the oral side of the obstructed segment. If necessary, the number of side openings at the tube’s end can be increased with aseptic scissors to boost drainage and decompression. Moreover, the side openings and the obstructed segment can be fixed to the tube to avoid slippage, as shown in **Figure 1**.
6. Drainage and Decompression: Connect the extension tube to an enema bag. Then, use lukewarm normal saline (37°C, 300 - 500 ml, administered hourly) for proper flushing and drainage.
7. Tube Removal: The tube is removed on the day of surgery or following the excision of the surgical specimen.

### 2.5 Outcomes

The primary outcomes cover alterations in obstructive conditions and adverse events subsequent to tube placement. The secondary outcomes involve intubation duration, the success of tube placement, as well as patient tolerance. The detailed specific outcomes are as follows:

1. Intubation: This includes the duration of the procedure, the number of intubation attempts, and the utilization of radiological fluoroscopy.
2. Effectiveness: To comprehensively evaluate the changes in intestinal obstruction, a series of parameters spanning symptoms, physical signs, laboratory tests, and imaging examinations are presented. Symptomatically, details such as the frequency and intensity of abdominal pain, the presence or absence of nausea and vomiting, and the restoration of normal bowel habits were monitored and recorded. For physical signs, particular focus was placed on the transumbilical abdominal circumference, abdominal distension, tenderness, and the detection of any abnormal masses. Laboratory tests furnished crucial data. Inflammatory markers, including C-reactive protein and white blood cell counts, along with electrolyte levels, were meticulously measured to reflect the body’s inflammatory response and internal equilibrium. Meanwhile, imaging examinations, such as abdominal X-rays or CT scans, provided visual evidence. They showed the degree of bowel dilation, the number of gas-fluid levels, the maximum transverse diameter of the proximally obstructed colon before and two days after tube placement, as well as any visible anatomical abnormalities within the colonic lumen. Additionally, information regarding intestinal dilation/edema and residual contents in the colonic cavity during surgery was also collected. These multi-faceted parameters jointly construct a comprehensive picture of the dynamic changes in intestinal obstruction.
3. Safety and Countermeasures: Tube-related discomfort symptoms following tube placement, tube blockage, displacement, or dislodgement; complications such as bleeding, perforation, rupture, infection, re-infarction, death, and other adverse events; and the administration of antibiotics or other medications related to the tube. These indicators were recorded from the initiation of tube placement until removal.

## 3 Results

### 3.1 Intubation process

Among the 42 patients, the procedure failed in 6 cases. The reason was that the obstructed segment had a tiny opening (i.e., ≤0.5 mm) or was completely blocked, which made it impossible to insert the guidewire under the endoscope. The remaining 36 cases were successfully intubated, achieving a single-attempt success rate of 85.7% (36/42). Among the 36 patients who had the tube successfully placed, 26 (72.2%) were male and 10 (27.8%) were female, with ages ranging from 50 to 88 years, and a median age of 61 years.

**Figure 2:**
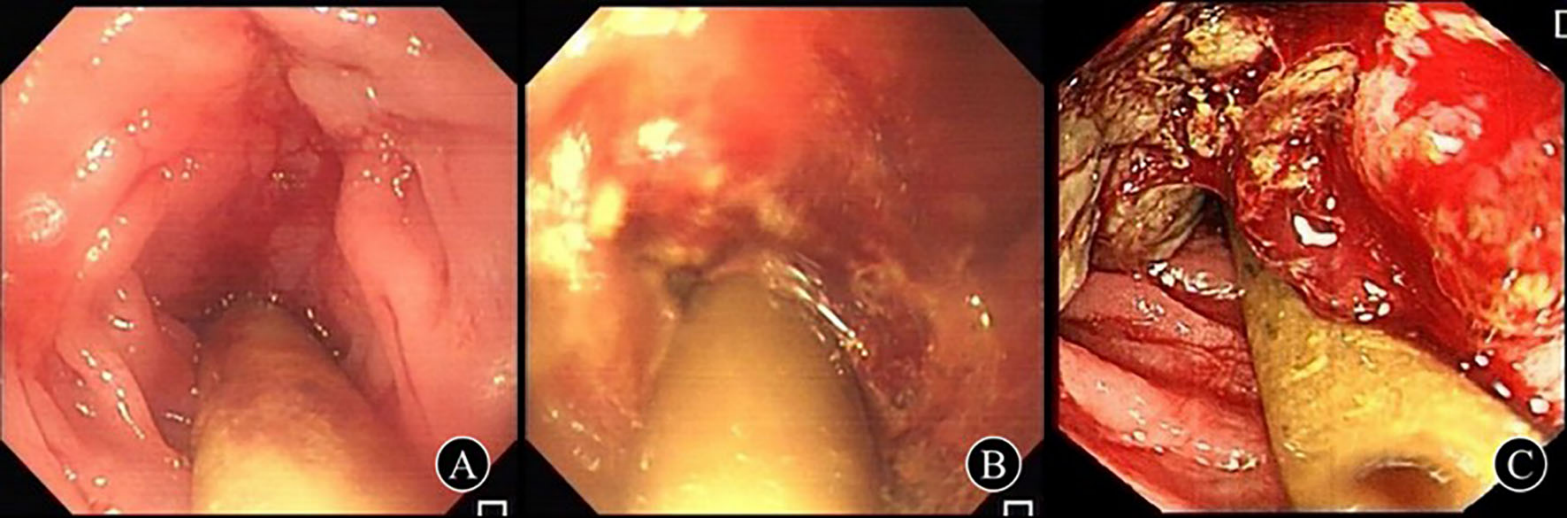
Status of the deeply inserted enema tube in obstructive segment of malignant left-hemicolon after successful placement **(A)** A 81-years-old female, 35 cm from the anus was found to have irregular uplift and depression in the circum-intestinal cavity, and the length of the obstruction was about 8 cm, which was diagnosed as descending colon cancer; **(B)** A 69-year-old male was diagnosed with rectal cancer due to circumferential luminal stenosis 10 cm from the anus, and the obstruction was about 5 cm long; **(C)** A 77-year-old male, 25 cm from the anus, was diagnosed as sigmoid carcinoma with periannular stenosis and obstruction about 6 cm long.

The distribution of the intestinal obstruction sites was as follows: 66.7% in the sigmoid colon, 25.0% in the rectum, and 8.3% in the descending colon. The distal end of the tube was deeply positioned within the intestinal lumen, roughly 10 - 15 cm closer to the proximal side of the obstructed segment. The average distance from the distal end of the tube to the anus was 37.4 ± 5.2 cm, while the mean length of the relieved obstructed segment was 8.2 ± 3.6 cm. Gastroscopy enabled successful intubation in only 6 cases (16.7%); for the remaining ones, an ultra-slim endoscope was used. Moreover, 2 cases (5.6%) necessitated X-ray fluoroscopy due to poor visualization when using the ultra-slim endoscope. The procedure took between 36 and 85 minutes, with an average duration of 47.1 ± 8.6 minutes. As depicted in **Figure 2**, the majority of this time was dedicated to bowel preparation.

### 3.2 Effectiveness evaluation

In the 36 patients with successful tube placement, the symptoms and signs of intestinal obstruction, namely abdominal pain, abdominal distension, vomiting, flatulence, and defecation, improved to varying extents. After 24 - 48 hours, the effective relief rate reached 100%. The time interval between successful tube placement and the onset of symptom improvement ranged from 1 to 5 minutes, with an average duration of 3.5 ± 1.2 minutes. The abdominal circumference decreased to a mean of (85.2 ± 3.0)% of the original value on the subsequent day (P < 0.01). On the abdominal plain film, both the amount of gas-fluid level and the maximum transverse diameter of the obstructed proximal colon decreased significantly [(4.5 ± 1.2) cm vs. (7.4 ± 0.8) cm, *P* < 0.01]. A downward trend was observed in CRP values. In 87.5% of the cases, CRP levels dropped by more than 50% within 48 hours, strongly suggesting a reduction in the inflammatory burden due to the obstruction. This decline was also correlated with the alleviation of clinical symptoms, such as the reduced frequency of abdominal pain, nausea, and vomiting. Comparison of key indicators before and after enema tube insertion was showed in **Table 1**. After 2 to 5 days (median 3), bowel sounds returned to normal in all patients. During surgery, there was slight or no obvious dilation and edema of the bowel, and no evident fecal residue was found in the colonic cavity.

**Table 1:** Comparison of key indicators before and after enema tube insertion.

Indicators	Ref.	Before	After	Statistics^△^	P
Abdominal circumference (cm)	80-90	98.2 ± 25.5	87.6 ± 12.7	t=2.105	0.039
Maximum transverse diameter of obstructed proximal left colon (cm)	2-5	7.4 ± 0.8	4.5 ± 1.2	t=11.374	<0.001
CRP (mg/L)	0-10	82.5 ± 17.1	35.8 ± 18.3	t=10.547	<0.001

△: pared-samples T test.

### 3.3 Safety evaluation

Following successful tube placement, 3 patients (8.3%) couldn’t tolerate the irritation from the tube and were thus referred for emergency surgery. In contrast, 14 patients (38.9%) had mild discomfort, which didn’t call for any specific intervention. During the tube placement, 6 cases (16.7%) reported abdominal pain and distension of varying intensities, mainly because of the tube flushing. Additionally, 2 cases (5.6%) each showed significant anal discomfort and minor rectal bleeding. Throughout the intubation process and the subsequent period, no serious tube-related adverse events took place, and no medications were given to address such issues in any of the cases.

### 3.4 Outcomes

During the period of tube placement, tube displacement occurred in 4 cases and obstruction happened in 1 case. All these incidents were successfully addressed with a single reintubation attempt. By the day of surgery, the average duration of tube placement was 10.8 ± 2.4 days. **Table 2** compares the peri-operative and post-operative outcomes between the clinically unsuccessful (n = 9) and successful (n = 33) tube insertion groups. The results indicated significantly higher intra-operative blood loss, prolonged operating time, and extended post-operative hospitalization in the unsuccessful group. Moreover, the unsuccessful group showed increased risks of post-operative complications and 3-month mortality.

**Table 2:** Comparison of peri-operative and post-operative outcomes between clinically unsuccessful and successful tube insertion groups.

Outcome	Unsuccess (n=9^*^)	Success (n=33)	Statistics^△^	*P* ^▽^
Emergency surgery / elective surgery^†^	7 (100%) / 0	0 / 32 (97.0%)	N/A	N/A
Resection and anastomosis	0 (0%)	28 (87.5%)	N/A	N/A
Enterostomy and/or resection	7 (100%)	4 (12.5%)	N/A	N/A
Intra-operative blood loss (mL)	330.8 ± 72.3	290.6 ± 40.4	t=2.047	0.048
Operating time (hours)	4.2 ± 2.8	2.8 ± 1.2	t=2.131	0.040
Post-operative complications^‡^	3 (42.9%)	2 (6.25%)	N/A	0.032
Post-operative hospital stays (days)	39.2 ± 3.5	29.8 ± 2.6	t=8.144	<0.001
Mortality within 3 months after discharge	3 (42.9%)	1 (3.13%)	N/A	0.014

*Containing 3 patients failed to tolerate the presence of the tube and still required emergency surgery; ^†^Missing medical records are attributed to hospital transfer or discharge against medical advice; ^‡^Abdominal infection, surgical site infection, anastomotic fistula, etc; N/A, not applicable; ^△^Independent-samples T test or Fisher's exact test; ^▽^two-tailed *P* value.

## 4 Discussion

We know that stent placement is also a treatment option for intestinal obstruction. However, this approach wasn’t the focus of our current study. The choice to use a deeply inserted drainage tube (enema tube) instead of a stent in our study was affected by multiple factors. Since our study was carried out in a developing country, considerations such as medical insurance coverage, the availability of stents, the technical proficiency local medical staff need for stent placement, cost-effectiveness, and the urgency of emergency situations all came into play. Drainage tubes are more accessible, less costly, and require relatively less specialized technical training to use, which makes them a more practical choice for our situation.

In this study, our objective was to attain a decompression effect by using a deeply inserted enema tube for the management of acute malignant left-sided colonic obstruction. Clinical observations indicated that, following successful tube insertion, the abdominal circumference had decreased to (85.2 ± 3.0)% on the second day (P < 0.01). On the abdominal plain film, the amount of gas-fluid level and the maximum transverse diameter of the obstructed proximal colon decreased significantly [(4.5 ± 1.2) cm compared to (7.4 ± 0.8) cm, P < 0.01]. CRP values exhibited a downward trend; in 87.5% of cases, they dropped by more than 50% within 48 hours, suggesting a reduction in the inflammatory burden caused by the obstruction. This decline was associated with the alleviation of clinical symptoms, such as less frequent occurrences of abdominal pain, nausea, and vomiting. All patients’ bowel sounds returned to normal within 2 - 5 days (median 3 days). During surgery, there was either slight or no bowel dilation/edema, and no conspicuous fecal residue was found in the colonic cavity. By the day of surgery, the average duration of tube placement was 10.8 ± 2.4 days. No serious tube-related adverse events were witnessed, and no medications were administered either during or after the tube insertion. Thus, the use of a deeply inserted enema tube is both feasible and safe for managing acute malignant left-sided colonic obstruction.

The progression of acute malignant left-hemicolon obstruction may lead to fluid loss, electrolyte disturbances, and acid-base imbalances. Moreover, severe infections, intestinal perforation, and death may arise due to intestinal ischemia and necrosis. At present, there is no standardized treatment for acute malignant left-hemicolon obstruction according to existing guidelines or consensus (7). In 1994, *Tejero* et al. were the first to employ metal stents for the management of this condition, achieving satisfactory results that provided essential transition time for patients awaiting elective surgery (8). In 2020, the European Society of Gastrointestinal Endoscopy (ESGE) explicitly endorsed self-expanding metallic stents (SEMS) as a bridge to elective surgery for acute left-sided malignant obstruction (9). The efficacy of metal stents in treating colorectal obstruction is well-established.

However, due to the material characteristics of metal stents, they are associated with a higher incidence of adverse events, including intestinal bleeding, perforation, and stent occlusion. Moreover, for patients with significant intestinal stenosis, the placement of metal stents may result in considerable discomfort that can be difficult to tolerate. Other notable issues encompass the limited availability of metal stents, their high cost, the necessity for specialized procedures, and their inability to be reused after displacement or removal. These factors further limit their application in numerous medical institutions in developing countries, such as China.

In recent years, the efficacy of ileus tubes and metal stents in the management of acute left hemicolon obstruction has gained substantial recognition (10, 11). Both ileus tubes and metal stents require placement under the guidance of a guidewire (12). However, studies have demonstrated that ileus tube placement is simpler, involves shorter operation times, and achieves higher success rates compared to metal stents. The decompression tube is equally effective in alleviating obstruction and offers the additional benefits of flushing the lumen and facilitating drainage. Furthermore, the ileus tube is more cost-effective, made from softer materials, and is generally better tolerated by patients. Moreover, it can be adjusted as needed and reused after displacement or removal (13). Nevertheless, the application of intestinal metal stents and ileus tubes in developing countries is constrained by challenges related to consumable management, high costs, the need for training, and potential complications.

In this context, we aimed to employ disposable enema tubes to address obstruction, drawing upon the applications of metal stents and ileus tubes, as well as the characteristics of enema kits, thereby achieving satisfactory results. Performing conventional colonoscopy and gastroscopy is extremely challenging in the intestinal lumen when visualization is compromised due to obstruction. In our study, the principal contributor to colonic obstruction was indeed stricture, with the situation varying across patients as not all colonic segments were fully occluded. Regarding endoscopic access, the colonoscope failed to pass through the strictures in all of our cases. Only in a handful of exceptional cases could the gastroscope make it through, while for the vast majority, we accomplished the procedure using an ultra-slim endoscope. Furthermore, two cases required X-ray fluoroscopy because of unclear visualization with the endoscope. Among the 42 patients, six cases failed due to a minimal opening (i.e., ≤0.5 mm) or complete closure of the obstructed segment, rendering it impossible to place the guidewire under the endoscope. The remaining 36 cases were successfully intubated, yielding a one-time success rate of 85.7% (36/42).

In such situations, even intestinal stents are likewise inapplicable (14). The distal end of the tube in the intestinal lumen was positioned approximately 10-15 cm proximal to the obstructed segment. The distance from the tube’s distal end to the anus averaged 37.4 ± 5.2 cm, with a relief length of the obstructed segment measuring 8.2 ± 3.6 cm. These parameters were comparable to those related to intestinal stents. Signs and symptoms related to intestinal obstruction improved to varying degrees following successful tube placement, achieving a relief rate of 100%. The interval between tube placement and symptom improvement ranged from 1 to 5 minutes, with a mean duration of 3.5 ± 1.2 minutes. These results demonstrate that the method is both effective and rapid, thereby fully addressing the therapeutic needs for intestinal obstruction decompression. To some extent, the decompression effect is comparable to that of intestinal metal stents or ileus tubes.

Since the material of the disposable enema tube is fundamentally similar to that of ileus tubes, we expect that they will demonstrate comparable tolerability. The results indicate that three patients (8.3%) were unable to tolerate tube stimulation and were subsequently referred for emergency surgery after successful placement, while 14 patients (38.9%) experienced minor discomfort that did not require specific intervention. This discomfort may be attributed to the relatively thick diameter and rigid composition of the disposable enema tube.

During the placement period, six cases (16.7%) exhibited varying degrees of abdominal pain and distension, primarily associated with tube flushing. Significant anal discomfort and minimal hematochezia were observed in two cases (5.6%). No serious tube-related adverse events occurred, and no medications were administered in any of the cases during or after intubation. By the day of surgery, the mean duration of tube placement was 10.8 ± 2.4 days. It can be concluded that endoscopic deep decompression using a disposable enema tube is safe and effective for the short-term management of acute left hemicolon obstruction, with transverse contrast comparable to that of an intestinal stent or ileus tubes (15).

Although disposable enema tubes and ileus tubes may be made from essentially the same materials, they are designed for distinct purposes. The disposable enema tube is primarily used for distal bowel irrigation, while ileus tubes are specifically designed for bowel obstruction. The primary advantage of the latter is that the tube tip features a balloon similar to that of a Foley tube. This design prevents the tube tip from slipping out of the obstructed segment, thereby ensuring continuous decompression.

Disposable enema tubes do not exhibit a comparable structural design. In this study, tube displacement was observed in four cases, while obstruction was noted in one case, resulting in a total of five cases during placement, all of which were successfully reintubated in a single attempt. To address this issue, a tube that is suitable for the diameter of the stenotic segment should be selected. If necessary, side holes may be created using sterile scissors (the quantity depending on the degree of obstruction) to enhance friction and assist in preventing slippage. This modification further enhances the effectiveness of subsequent irrigation and drainage.

To maintain sustained decompression, subsequent tube maintenance and irrigation are crucial. In this context, we summarize our practical experience as follows: Following successful drainage, the symptoms and signs of obstruction should be monitored on the same day. Additionally, the negative pressure suction device should be connected to maintain continuous low negative pressure, while intermittent irrigation should be performed based on drainage patency. This requirement stems from the accumulation of gas and fluid in the intestinal lumen during the initial phase of decompression. In the absence of any potential blockage within the lumen, routine saline irrigation is generally not required.

Subsequently, saline irrigation was initiated based on the consistency and drainage characteristics of the stool. The volume of irrigation was approximately 300 to 500 mL per hour. Simultaneously, the symptoms and signs of the patients were monitored, along with their intake and output, to maintain homeostasis and ensure that output exceeds intake. Poor drainage represents an inherent challenge. An appropriate volume of laxatives or hypertonic saline may be administered through the tube. This approach aids in enhancing the osmotic pressure within the intestinal lumen, reducing edema of the intestinal mucosa, and facilitating the cleansing and elimination of bacteria. Concurrently, intermittent suction should be utilized.

When necessary, a guide wire is inserted to facilitate minor adjustments in the tube position. Extensive irrigation is contraindicated. While monitoring intake and output, it is essential to assess the patient’s electrolyte and acid-base balance, along with other internal environmental factors, and to provide appropriate supportive treatment. If abdominal pain occurs, complications such as bleeding and perforation should be prioritized for evaluation. Furthermore, the possibility of drainage blockage must be considered. For abdominal or anal discomfort, diclofenac sodium may be administered rectally, or a compounded formulation of carrageenan and lidocaine may be applied locally to alleviate discomfort.

The main benefit of this therapy, as our study demonstrated, is indeed to reduce the need for emergency surgeries and to buy time for elective procedures. In our retrospective case series, we frequently encountered patients with complex bowel obstructions who were in poor general condition. Through this treatment approach, we were able to stabilize their condition, alleviating acute symptoms and allowing for more comprehensive preoperative preparations. This not only improved the patients’ tolerance to surgery but also potentially reduced the associated surgical risks. However, we acknowledge that our study was designed as a retrospective case series, and regrettably, we did not have a control group with stent placement as a bridge to surgery. Due to the nature of data collection in this retrospective setting, we were limited in our ability to directly compare outcomes between different treatment modalities. Nevertheless, the outcomes of the decompressed patients in our series were quite encouraging. Among them, 85.7% of patients successfully underwent elective resection and anastomosis after a period of decompression, avoiding the higher morbidity associated with emergency operations.

In summary, malignant obstruction of the colon presents a considerable clinical challenge. Although the use of intestinal stents and ileus tubes for treating malignant obstruction of the left colon has yielded recognized results, their widespread adoption is hindered by various factors, particularly in developing countries. This study demonstrates that endoscopic decompression using a disposable enema tube is both feasible and safe for treating acute malignant left-hemicolon obstruction. It is easily accessible, straightforward to operate, and cost-effective, rendering it a viable emergency alternative for developing countries. Given the small sample size and the short-term retrospective nature of this study, further high-quality research designs and follow-up assessments are necessary to obtain more reliable clinical evidence regarding the effects of endoscopic deep decompression with a disposable enema tube on elective surgery outcomes and long-term survival.

## Data Availability

The original contributions presented in the study are included in the article/supplementary material. Further inquiries can be directed to the corresponding authors.
